# Supplementary material for: Effects of Bacterial CLPB Protein Fragments on Food Intake and PYY Secretion
Source: Nutrients. 2021 Jun 29;13(7):2223. doi: 10.3390/nu13072223 (PMC8308458; doi:10.3390/nu13072223)
Supplement: Supplementary file 1 [file nutrients-13-02223-s001.zip › nutrients-1224440-supplementary.pdf]

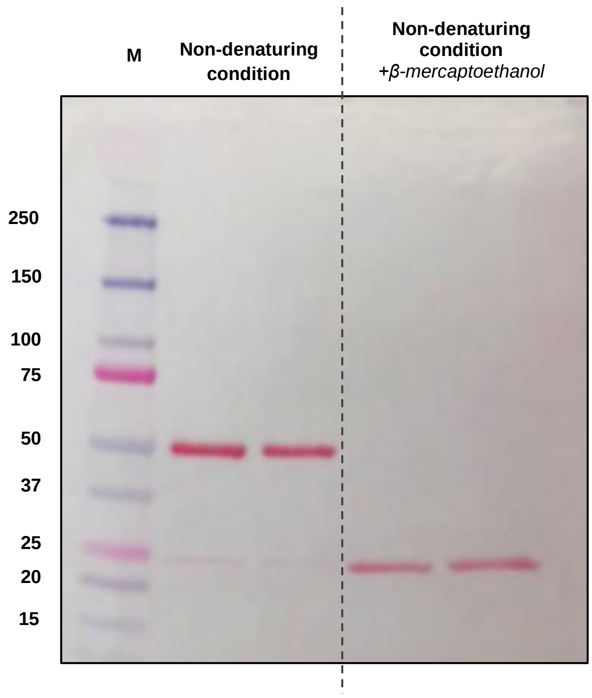


**Supplemental Figure 1. Verification of ClpB25[1] purification by Western Blot.**
Western Blot of purified ClpB25 in non-denaturating conditions (without β-mercaptoethanol) was showed the presence of two bands at 50 kDa and 25 kDa after a revelation by a Ponceau red coloration. ClpB50 was higher intensity that ClpB25. *A contrario*, Western Blot of purified ClpB25 in denaturating condition (with β-mercaptoethanol) revealed only a ClpB25.

[1] CLPB25, Location 536-756aa on sequence UniProtKB-P63284 CLPB_ECOLI.

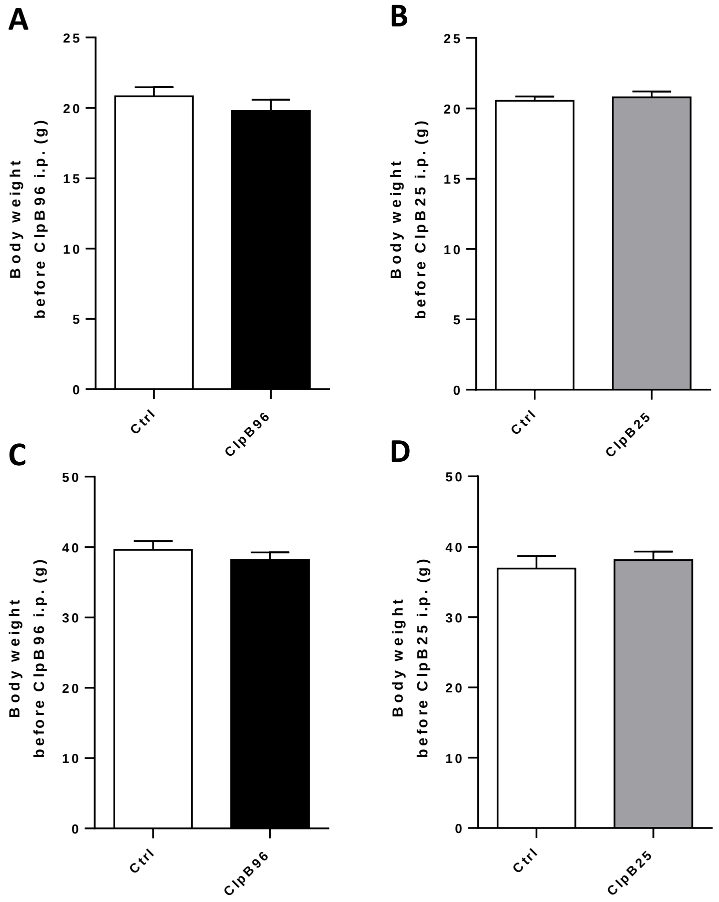


**Supplemental Figure 2. Body weight of mice before injection of CLPB96 or CLP25 in (A) C57Bl/6 or (B) ob/ob mice.** (A), n=8. (B), n=6 for Ctrl and n=10 for CLPB96 or CLPB25. CLPB96, recombinant CLPB protein: UniProtKB-P63284 CLPB_ECOLI. CLPB25, 25 kDa CLPB fragment: Location 536-756aa on sequence UniProtKB-P63284 CLPB_ECOLI.
